# A Case of Severe Refractory Pemphigus Vulgaris in a Patient With Stable Esophageal Malignancy

**DOI:** 10.7759/cureus.14576

**Published:** 2021-04-20

**Authors:** Vanessa C Browne, Catherine Choi, Eugenio M Capitle, Reena Khianey

**Affiliations:** 1 Internal Medicine, Rutgers University, Newark, USA; 2 Rheumatology, Rutgers University, Newark, USA

**Keywords:** pemphigus vulgaris, paraneoplatic pemphigus, esophageal cancer, bollous autoimmune diseases, desmoglein, auto immune, autoantigens, autoantibodies, intravenous immunoglobulins (ivig), plasmapheresis

## Abstract

Pemphigus is a broad term that is used to describe a group of bullous autoimmune diseases affecting the skin and mucous membranes; the pathogenesis involves autoantibodies directed against various cell junction desmosomal proteins. In patients with a history of malignancy who present with bullous lesions, the differential diagnosis may include, but is not limited to, paraneoplastic pemphigus (PNP) and pemphigus vulgaris (PV) secondary to a primary autoimmune process, or induced by chemotherapy or radiation therapy. In this report, we present a case of refractory PV in a patient with stable esophageal cancer, five years after undergoing radiation therapy. He was poorly responsive to corticosteroids and intravenous immunoglobulin (IVIG). PNP or PV in a patient with stable esophageal malignancy has not been previously reported. PNP and PV can have overlapping autoantigens [desmoglein types 1 and 3 (DSG1 and DSG3)] as well as similar presentations. Thus, distinguishing between the two may be challenging in a patient with a history of cancer. More research must be done to determine if PNP can be seen in a patient with stable esophageal malignancy and, similarly, if PV can be precipitated by stable esophageal malignancy. Such research would aid in determining whether or not similar presentations are more severe or refractory to standard treatment regimens, thereby contributing to improve treatment strategies.

## Introduction

The broad term pemphigus refers to a group of bullous autoimmune diseases that affect the skin and mucous membranes [1]. The pathogenesis of pemphigus diseases involves autoantibodies directed against various desmosomal proteins; these proteins form junctions between neighboring cells and are therefore important for the architecture and mechanical strength of tissues [1]. Pemphigus diseases can be divided into four major subtypes: pemphigus vulgaris (PV), pemphigus foliaceus, paraneoplastic pemphigus (PNP), and immunoglobulin A (IgA) pemphigus [1]. PNP in a patient with stable esophageal malignancy and PV precipitated by stable esophageal malignancy have not been previously reported. PNP and PV can have overlapping autoantigens [desmoglein types 1 and 3 (DSG1 and DSG3)] as well as similar presentations, and hence distinguishing between the two may be difficult in a patient with a history of cancer. We report a case of refractory PV in a patient with stable esophageal cancer, five years after undergoing radiation therapy. The patient was poorly responsive to corticosteroids and intravenous immunoglobulin (IVIG).

## Case presentation

**Figure 1:**
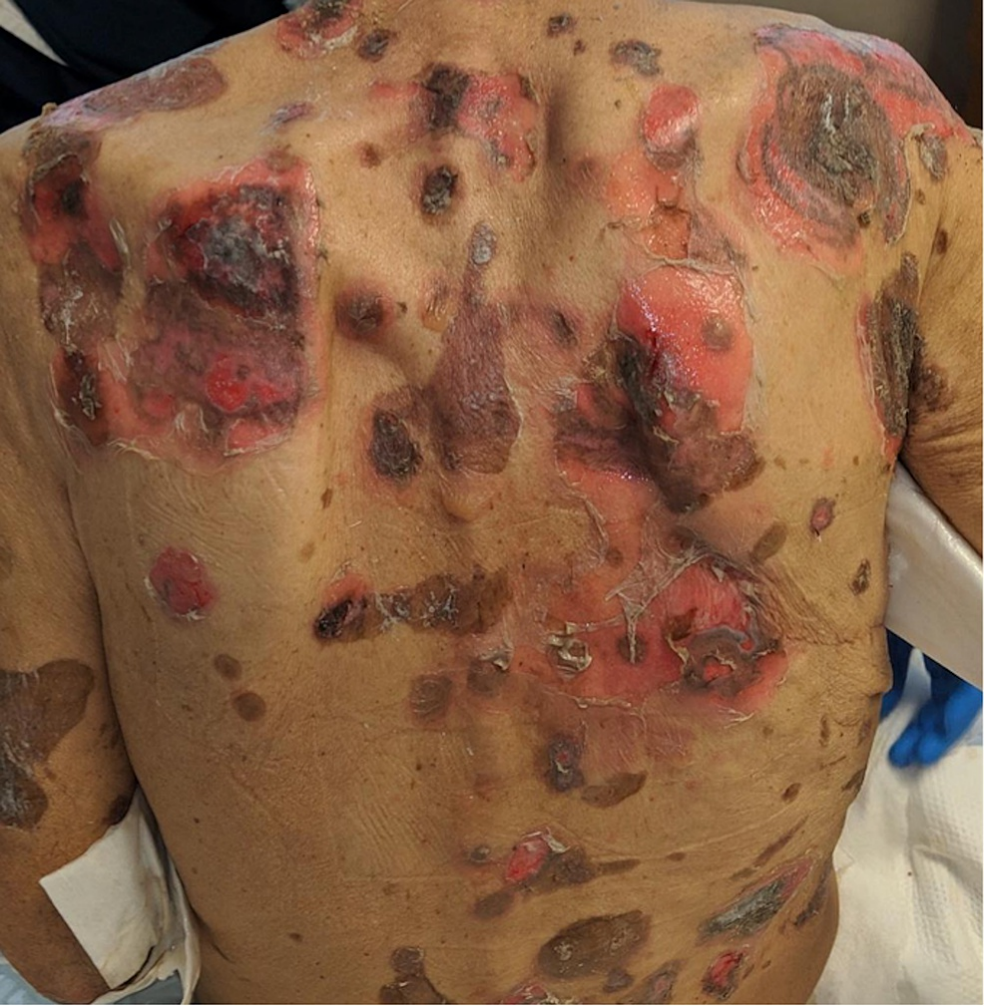
Photo of the patient at presentation Extensive flaccid bullae subsequently turned into ruptured blisters and deep erosions on the back and arms

A 76-year-old man with a history of stage IIB (T2N1M0) squamous cell carcinoma of the distal esophagus, cerebrovascular accident, type 2 diabetes mellitus, and hypertension presented to the emergency department with a bullous disease affecting 70% of his body. His symptoms had initially started five months prior to the presentation, with oral ulcers and dysphagia; three months later, bullae had appeared on his arms and had later spread to his torso and legs. Thereafter, the bullae had begun to open, causing erosions, and ulcerations with erythematous borders and crusting. Of note, he had been diagnosed with squamous cell carcinoma of the distal esophagus seven years prior and undergone radiation therapy, neoadjuvant chemotherapy with cisplatin, and subsequent esophagectomy; he had been in remission for five years. He denied starting any new medications, and CT chest/abdomen/pelvis two weeks prior to the presentation had shown no evidence of malignancy. A review of systems was positive for a 100-lb weight loss over the preceding five months but was negative for fever, chills, night sweats, chest pain, and shortness of breath. On physical exam, oral mucosal ulcers and diffuse tense skin bullae affecting 70% of body surface area were noted (Figure 1). Rheumatology was consulted due to concern for PV and for a skin biopsy.

Complete blood count (CBC), complete metabolic panel (CMP), and complement components (C3 and C4) were all within normal limits. However, erythrocyte sedimentation rate (ESR) was elevated to 36 mm/hour and C-reactive protein (CRP) was elevated to 25 mg/L. The investigation for drug allergy was negative and, initially, his infectious workup was also negative. Right buccal mucosal biopsy with immunofluorescence demonstrated IgG and C3 in the squamous intercellular zone. Right thumb biopsy with immunofluorescence demonstrated a detached fragment of the superficial epidermis and a detached fragment of the necrotic epidermis with features of acantholysis and an intercellular pattern in the epidermis with IgG and IgM (weak). Both studies were consistent with PV. Upper endoscopy was performed to rule out the recurrence of esophageal cancer. Two small esophageal nodules were found and both revealed findings consistent with PV but not malignancy. CT chest/abdomen/pelvis was obtained due to concern for pemphigus as a paraneoplastic presentation given his history of esophageal squamous carcinoma. There was no convincing evidence of cancer recurrence on CT.

The patient was initially started on pulse dose steroids, which were later held as methicillin-sensitive *Staphylococcus aureus* (MSSA) bacteremia was confirmed with blood cultures, and he was subsequently started on cefazolin. He was then started on colchicine, which was discontinued after four days due to the progression of illness characterized by new diffuse bullae involving 90% of his body surface area. He was restarted on IV steroids and given two rounds of IVIG with minimal improvement. He then received two doses of rituximab with some improvement, as well as seven cycles of plasma exchange. The patient was subsequently discharged on a steroid taper. A significant resolution of the mouth ulcer and skin bullae was noted at his six-week outpatient follow-up appointment.

## Discussion

Cutaneous paraneoplastic syndromes were initially suspected in our patient due to his age and significant cancer history. PNP is a rare subtype of the pemphigus group; it is most often associated with lymphoproliferative neoplasms such as non-Hodgkin's Lymphoma, Castleman disease, and chronic lymphocytic leukemia [1,2]. However, there have been reports of PNP in non-hematological neoplasms [2]. PNP involves four accepted clinical features: stomatitis, acantholysis/dermatitis, antiplakin antibodies (DSG1 and DSG3), and an underlying neoplasm [1,2]. In a typical case of PNP, the neoplastic process is well established at the time of the onset of skin manifestations indicative of PNP [3]. Four cases of PNP with associated esophageal cancers have been reported thus far, and all of them had active malignancy at the time of diagnosis of PNP [4]. Although this patient had a history of squamous cell carcinoma of the distal esophagus, further workup demonstrated no evidence of active malignancy. Hence, PNP was placed lower on the differential as per more diagnostic test results, although we did not completely rule it out since PNP and PV can have overlapping autoantigens [2,5]. The management of PNP includes treatment of the malignancy, and the first-line therapy is usually high-dose systemic glucocorticoids [6].

PV involves IgG antibodies directed against DSG1 and/or DSG3 leading to endocytosis of these proteins, thereby causing failure of desmosome assembly [2,5]. As a result, the architecture and strength of epithelial tissue are disrupted, leading to a bullous disease [2]. PV can be further divided into mucous-predominant and mucocutaneous forms. Mucosal-dominant forms usually only involve antibodies against DSG3, whereas the mucocutaneous form involves both DSG1 and DSG3 [5].

PV often presents as extremely painful erosions on mucosal membranes, and therefore can significantly impair the quality of life of patients. Moreover, mortality among patients with PV is high due to the risk of infections, especially pneumonia and sepsis [1]. In studies conducted in Israel, the infectious cause of death was reported to be 22.6 fold higher in patients with PV compared to the matched general population [1]. This is largely because patients with PV require continuous use of corticosteroids and immunosuppressive therapy as part of their treatment. This was also true in our patient who had a prolonged hospital stay due to complications of MSSA bacteremia. Fortunately, this patient survived his hospital course; however, his bacteremia necessitated the halting of treatment for PV during which time his condition worsened.

PV can be associated with malignancy. Kridin et al. performed a cross-sectional study involving 11,859 participants to determine whether or not there is an association between pemphigus and solid malignancies. They compared the prevalence of 17 different solid malignancies among patients diagnosed with PV or pemphigus foliaceus. They found a statistically significant association between esophageal cancer as well as laryngeal cancer with pemphigus [7]. That being said, in this study, the participants appeared to have active malignancy, or at least the authors did not differentiate between inactive or active malignancy. Our patient had inactive esophageal cancer that had been in remission for several years, and hence it is unclear if his PV was associated with his inactive malignancy. Therefore, more studies would need to be performed to determine the association of PV with inactive malignancies.

PV secondary to chemotherapy was also considered in this patient since new autoimmune diagnoses have been reported in patients after receiving chemotherapy. Thyroid disease, colitis, and type 1 diabetes mellitus have all been reported in patients after undergoing treatment with checkpoint inhibitor therapy [8]. When our patient had been diagnosed with squamous cell cancer of the esophagus, he had undergone neoadjuvant chemotherapy with cisplatin. Cisplatin is a platinum-based medication that forms covalent bonds with DNA bases, impairing crosslinking within the double helix and thereby DNA synthesis [9]. Though cisplatin is not a checkpoint inhibitor, some case reports have described patients developing autoantibodies after receiving various chemotherapy regimens, not just checkpoint inhibitors; however, the mechanisms are still being investigated and little is known about their clinical significance [10].

PV is largely an idiopathic condition; however, radiation-induced PV (RIPV) cases have been reported as well, though only a few. In one case series, all three cases of RIPV occurred within 14 months of radiation therapy, and symptoms initially occurred in areas where radiation was given and eventually disseminated to other areas of the body [11]. There are several proposed mechanisms as to why some patients may develop PV after receiving radiation therapy. One such mechanism involves surface proteins on keratinocytes being altered, thereby changing the antigenicity of the cell, leading to the development of antibodies [11]. Our patient presented five years after undergoing radiation therapy; it is unclear if RIPV can still occur after such a long period following radiation. More cases would need to be documented and studied to determine the extent and duration of risk after radiation therapy and other factors involved in this disease process. RIPV is often responsive to systemic glucocorticoids [12]. However, our patient had a poor response to steroids and IVIG. His condition finally improved with rituximab and plasmapheresis.

## Conclusions

PV and PNP can have overlapping autoantigens and hence can be difficult to differentiate in patients with malignancy. PNP in a patient with stable esophageal cancer has not been reported yet, although there have been four reported cases of PNP in patients with active esophageal malignancy. In summary, underlying autoimmune-associated PV should be distinguished from PNP as well as PV as an adverse effect of radiation therapy or chemotherapy. This is because each of these conditions may require distinct prognoses and treatment methods that differ from each other. Further studies are warranted to determine whether or not PNP or PV can be precipitated by esophageal cancer that has been in remission for several years.
